# Supplementary material for: Synthesis and Characterization of Porous MgO Nanosheet-Modified Activated Carbon Fiber Felt for Fluoride Adsorption
Source: Nanomaterials (Basel). 2023 Mar 16;13(6):1082. doi: 10.3390/nano13061082 (PMC10051765; doi:10.3390/nano13061082)
Supplement: Supplementary file 1 [file nanomaterials-13-01082-s001.zip › nanomaterials-2296751-supplementary.pdf]

Synthesis and characterization of porous MgO nanosheets modified activated carbon fiber felt for fluoride adsorption

De-Cai Wang [a,b], Min-Da Xu [b], Zhen Jin [a,b*], Yi-Fan Xiao [c], Yang Chao [a,b], Jie Li [a,b], Shao-Hua Chen [a,b], Yi Ding [a,b*]

a. Anhui Advanced Building Materials Engineering Laboratory, Anhui Jianzhu University, Hefei 230601, Anhui, China.

b. School of Materials and Chemical Engineering, Anhui JianZhu University, Hefei Anhui 230601, PR China.

c. School of Environment and Energy Engineering, Anhui Jianzhu University, Hefei Anhui 230601, PR China.

*Correspondence should be addressed to Zhen Jin and Yi Ding

E-mail: ftbjin@hotmail.com and dyrqf@ahjzu.edu.cn

Tel.: +86-551-63828103

Supplementary Material

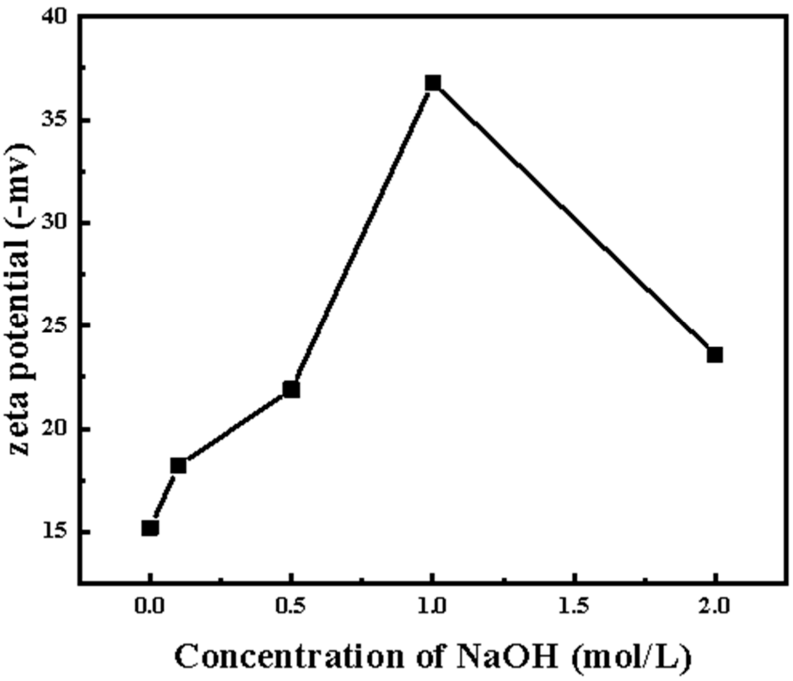

Figure S1 illustrates the Zeta potential of the ACFF treated with different NaOH solutions.

After we determined that the use of NaOH solution could activate ACFF surface to make it easier to modify MgO on load, Zeta potential was used to determine the optimal concentration of ACFF activation with NaOH solution. It can also be observed from Fig. S1 that the Zeta potential of ACFF under the treatment of 1mol/L NaOH solution is the highest, indicating that the negatively charged hydroxyl functional groups on the surface of ACFF reach a peak and the number of sites that can bind with MgO also reaches a peak.

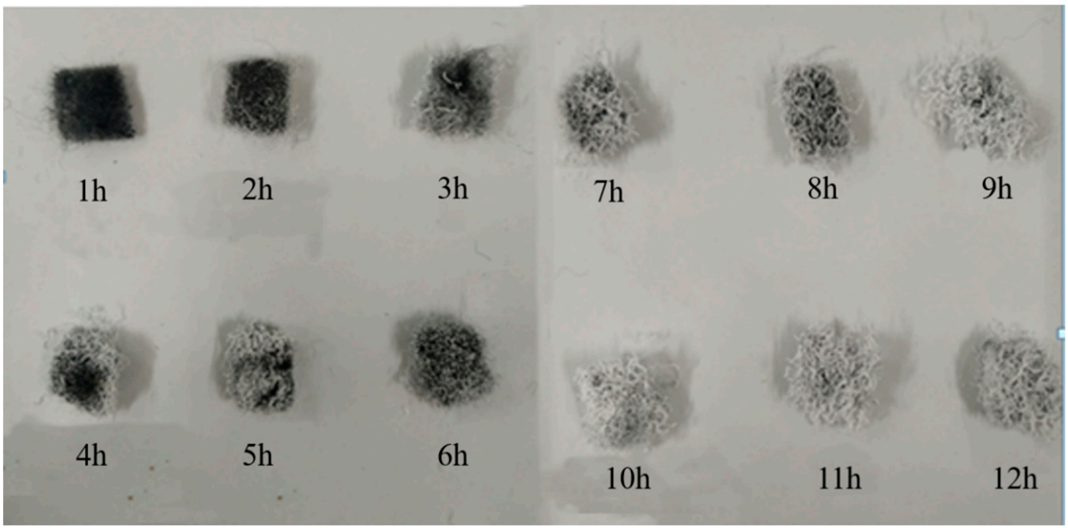

Figure S2 the digital images of the MgO modified ACFF with different reaction time.

Figure S2 shows the physical diagram of ACFF load of MgO at different hydrothermal times, from which it is obvious that ACFF load is gradually increasing. The 12h reaction has made MgO grow well on the surface of ACFF, and the exposed ACFF is almost invisible to the naked eye.

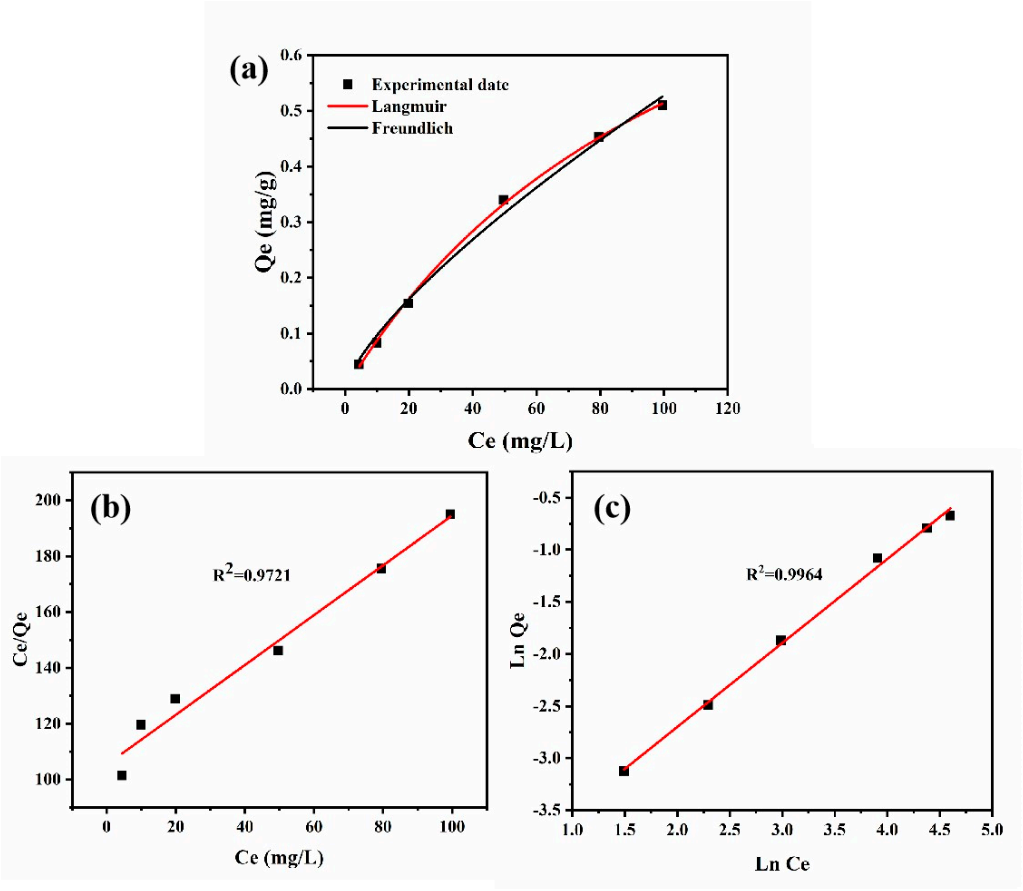

Figure S3(a) Adsorption isotherm of fluoride on the ACFF (b) the corresponding linear fitting of Langmuir model; (c) the corresponding linear fitting of Freundlich model.

Figure S3 studied the adsorption capacity of ACFF in blank group under different concentration of initial fluorine solution, and the Langmuir and Freundlich models were fitted to it. ACFF was more consistent with the Freundlich model, indicating the multilayer adsorption performance of ACFF and its low binding ability to fluoride ion. It also highlights the performance improvement of MgO loaded ACFF.

Table S1 Pseudo-second-order model parameters for fluoride removal on the MgO@ACFF

$q_{e(theo)}$(mg/g)	k_2 (g $mg^{-1}min^{-1}/10^{-4}$)	R^2	$q_{e(exp)}$(mg/g)
95.97	2.90	0.9991	94.36

Table S1 gives the parameters of MgO@ACFF under the second-order kinetic fitting in detail. The fitting degree is quite high, and the theoretical maximum adsorption capacity of fluoride ion in the fluoride ion solution of 100mg/L can reach 95.97mg/g, showing the excellent chemical adsorption performance of MgO@ACFF.
